# Preliminary Study of Pepper Types Based on Multielement Content Combined with Chemometrics

**DOI:** 10.3390/foods12163132

**Published:** 2023-08-21

**Authors:** Michaela Zeiner, Heidelore Fiedler, Iva Juranović Cindrić, Ivan Nemet, Doris Toma, Iva Habinovec

**Affiliations:** 1 Man-Technology-Environment Research Centre, School of Science and Technology, Örebro University, Fakultetsgatan 1, 70182 Örebro, Sweden; heidelore.fiedler@oru.se; 2 Department of Chemistry, Faculty of Science, University of Zagreb, Horvatovac 102a, 10000 Zagreb, Croatia; ijuranovic@chem.pmf.hr (I.J.C.); inemet@chem.pmf.hr (I.N.); ihabinovec@chem.pmf.hr (I.H.)

**Keywords:** chemometric analysis, ICP-MS, major and minor elements, pepper types, spice characterization

## Abstract

Different types of pepper (*Piper nigrum* L.) and cayenne pepper (*Capsicum annuum* L.) are widely used spices that exhibit therapeutic properties in addition to nutritional properties. In order to characterize these foods in further detail, the content of macro- (Ca, K, Mg, Na) and microelements (Ag, Al, As, Ba, Be, Bi, Cd, Co, Cr, Cu, Fe, Ga, Li, Mn, Mo, Ni, Pb, Rb, Se, Sr, Te, Tl, V and Zn) of four pepper types was determined via inductively coupled plasma mass spectrometry (ICP-MS) after microwave-assisted digestion using nitric acid and hydrogen peroxide. The obtained results were then evaluated using chemometric methods. The content of macroelements and microelements lies in the expected ranges for such spices but differs significantly between different types. The content of macro- and microelements is characteristic for pepper types originating from different plant species, but also based on further processing. Whilst green and black pepper are similar to each other, clearly diverse patterns are obtained for white pepper (different processing method) and cayenne pepper (different plant species).

## 1. Introduction

Pepper has high economic, commercial and medicinal values and is commonly used in cooking and as a seasoning. Black, white, and green pepper are all derived from the same plant, *Piper nigrum*, but they are processed differently and thus have different characteristics. The distinctive pungent flavor of black pepper is mainly due to the alkaloid called piperine and its structural analogues [1,2,3]. Green pepper has a milder flavor than black or white pepper. White pepper is derived from fully ripe fruits of the *Piper nigrum* plant by removing the outer pericarp before drying. Green pepper is based on unripe fruits harvested approx. 10–15 days before maturation which are processed in such a way that the green color is maintained. Cayenne pepper, however, is made from the dried, ground fruit of several species of chili peppers, including *Capsicum annuum* and *Capsicum frutescens*. It has a bright red color and a very hot, spicy flavor. Plant species do contain varying amounts of metals and metalloids, a fact that can be used to help identify different plant species. The metal content of a plant can vary depending on factors such as soil composition, climate, and growing conditions [4].

The production and distribution of high-quality spice is an important issue in the food industry. The quality of pepper can be determined and checked, for example, via physical and chemical parameters, such as foreign matter, empty grains, density, moisture, composition of ether extract, contents of total ash, protein, crude fiber and piperine [5]. In order to ensure a safe use, attention needs to be paid to imported spices regarding toxic metal contaminations [5,6,7]. One way that metal content can be used for identification is through the analysis of plant tissue samples or derived products, e.g., processed food products. Researchers can measure the contents of different metals and use this information to help identify the species of plant. This type of analysis is often used in studies of plant ecology, environmental pollution, bioremediation, and for food authentication [7,8,9,10,11,12,13]. Overall, the metal content of plants can provide important information about their identity and ecological function and can be a useful tool in the study of plant biology, ecology and food industry. During the past two decades, the consumption of botanical products has increased as result of the consumers’ trend to focus on and use more natural and high-quality botanical products [7,8,9,10,11,12,13].

Inductively coupled plasma mass spectroscopy (ICP-MS) is a well-established method for simultaneous multi-elemental analysis of a wide range of (trace) elements in digested food samples and has thus been chosen for this study [9,10,13,14,15,16].

The application of multivariate analysis for quality control and authentication of food products including spices and herbs has increased over the last 10 to 15 years. Combining spectrometric techniques with multivariate chemometric tools offers more options to evaluate element contents [9,10,17,18]. 

The present investigation aimed to, firstly, analyze different pepper samples commercially available on the Croatian market since pepper is known as a spice that significantly contributes to healthy nutrition, and, secondly, to determine whether the obtained elemental pattern can be used to discriminate different pepper species. The methodology applied was acidic microwave-assisted digestion followed by ICP-MS analysis, with a focus on the following elements: Ag, Al, As, Ba, Be, Bi, Ca, Cd, Co, Cr, Cu, Fe, Ga, K, Li, Mg, Mn, Mo, Na, Ni, Pb, Rb, Se, Sr, Te, Tl, U, V and Zn. Comparing metal contents in different kinds of pepper leads to useful information for scientific disciplines related to diet. 

## 2. Materials and Methods

### 2.1. Samples, Chemicals and Material

The pepper samples (dried fruit) were acquired in March 2020 from markets in Zagreb, Croatia. They were imported from India and packed in plastic bags (15 g each). Two different batches of each sample type were bought. The certified reference material (CRM) of strawberry leaves, from LGC Standards (United Kingdom; LGC7162), was used for quality control/quality assurance.

Supra pure nitric acid (HNO_3_), hydrogen peroxide (H_2_O_2_) of p.a. (pro analysis) quality were acquired from Merck (Darmstadt, Germany). The stock solution used for the preparation of the calibration standards in concentrations ranging from 0.01 µg/L to 100 µg/L, namely ICP Multielement Standard IV (1000 mg/L), was from the same company. All glass- and plastic-ware were pre-cleaned with semi-concentrated nitric acid prior to use. Ultrapure water (>18 MΩ cm) used during the entire study came from the in-house production unit. 

### 2.2. Sample Preparation and Chemical Analyses

After arrival at the laboratory, the samples were homogenized in a metal-free mortar and stored in a dry and dark room at ambient temperature prior to digestion. In triplicates, 0.2 g portions (weighed to the nearest 0.1 mg) of dry, ground and sieved pepper samples were mixed with 6 mL nitric acid (50:50 *v*/*v*) and 6 mL hydrogen peroxide (30%) in Teflon digestion vessels and the digestion was continued slowly at room temperature for approximately 3 h. Afterwards, the sample underwent microwave treatment (MWS-2 Speedwave Berghof), followed by heat treatment and power, namely 30 min at 120 °C (500 W), 30 min at 170 °C (700 W), and 30 min at 130 °C (400 W). The obtained solutions were left to cool until they reached room temperature, and then transferred to the volumetric flasks and to a constant volume (25 mL) with deionized water. Prior to use, the Teflon digestion vessels, were soaked for 24 h with 2% (*v*/*v*) nitric acid and then rinsed with deionized water to avoid unexpected contaminations during the sample preparation. The applied sample pretreatment procedure is characterized by its easy use in addition to its ability to minimize contamination and experimental errors by reducing, first, the number of different reagents and, later, interferences during the measurements. Elemental analysis of the samples via ICP-MS (Agilent 7500cx ICP-MS, Agilent, Tokyo, Japan) was performed after a preceding microwave-assisted digestion step. In this work, nitric acid and hydrogen peroxide were used to decompose the pepper sample matrix.

### 2.3. Figures of Merit of the Analytical Method

The CRM consisted of strawberry leaves used to prove the accuracy, i.e., precision and trueness, of the applied method. Analytical quality control was performed through spiking experiments at two selected concentrations for the elements not certified in the CRM. Precision as intra-day precision was expressed as relative standard deviation based on ten replicates of one sample (CRM or spiked digest solution). Trueness was expressed as recovery (percentage found) either referring to the CRM or to the spiking experiments. The limits of the detection of the elements were based on three times the respective standard deviation.

### 2.4. Data Treatment and Statistical Methods

The data were stored in Microsoft Office 365 Excel^®^ (Microsoft 365, version 2305, Microsoft Corporation, Redmond, WA, USA). To perform statistical evaluations and visualization, R packages (versions 4.0.3 and 4.0.5) with R-Studio (version 2022.12.0+353, Posit Software, PBC, Boston, MA, USA) were utilized. Multivariate methods, specifically hierarchical cluster analysis (HCA) and principal component analysis (PCA), were employed to assess the similarities, differences, or correlations between datasets and meta-data, referred to as “Type”.

Clustering was carried out using Euclidean distances and the Ward method (ward.02 in R), which creates groups with minimized variance within the clusters. This approach grouped the data into clusters of higher similarity, forming a dendrogram as shown in the heatmap (refer to Section 3.3). In PCA, the data were extracted and projected to display systematic variations in a data matrix. For all quantitative assessments, concentrations below the limit of quantification (LOQ) were considered to be zero. 

To determine whether there were statistically significant differences between the independent and dependent variables, non-parametric testing was performed using the Kruskal–Wallis H test. Post hoc analysis was conducted using the pairwise Wilcoxon test. Adjustment of the values was carried out using the Benjamini–Hochberg method, and the significance level was set to *p* = 0.05. Additionally, the correlation between variables was assessed using the Pearson method.

## 3. Results

### 3.1. Method Characterization

The method used was fully validated in a previous study [16], and in the present study, the method was checked by analyzing the CRM of strawberry leaves. The obtained validation data (trueness) justify the usage of external calibration based on non-matrix-matched standard solution. All calibration curves have coefficients of determination of *R*^2^ > 0.997. In this study, only the elements found, i.e., with concentrations above their respective limits of detection (LOD), were selected as variables for the following chemometric calculations. The LODs, recovery in % and precision are given together with the obtained results in Tables S1 and S2. The overall uncertainty of measurement of the applied analytical procedure was determined to be <9% for all analytes considering a coverage factor of k = 2.

### 3.2. Elemental Composition of Peppers

**Figure 1:**
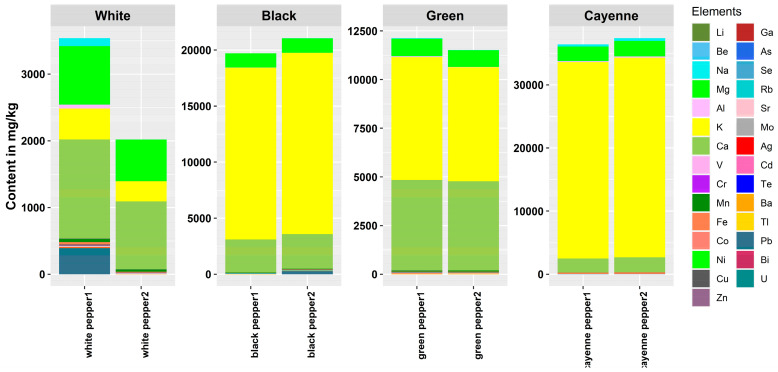
Elemental pattern of four pepper types.

The elemental pattern of the analyzed pepper samples is depicted in Figure 1 as the sum of all determined elements, as well as presented as a percentage composition. Detailed information of all analytes including the validation parameters (LOD, precision, recovery) is given in the supplementary information in Tables S1 and S2.

The results of the elemental analysis of pepper samples indicate that K, Ca, and Mg were the most abundant macroelements, followed by Na. The highest content of macroelements was found in cayenne pepper and the lowest in white pepper. From these results, it is apparent that the macroelements differ with respect to the species and way of processing. The order K, Ca, Mg, and Na was also found for black pepper samples originating from Tunisia [19].

The content of K (mean value 13,394 mg kg^−1^) is in a similar range to that found in literature data (25,850 mg kg^−1^–26,920 mg kg^−1^, [9] and 13,960 mg kg^−1^ and 20,770 mg kg^−1^, [12]) but even the lowest content found in white pepper (383 mg kg^−1^) is higher than the results obtained by Savić et al. (112 mg kg^−1^, [20]). 

After K, the highest content is detected for calcium. Ca was found in a range from 1075 mg kg^−1^ to 4614 mg kg^−1^. Savić et al. found a higher value of Ca in black pepper (3968.79 mg kg^−1^, [20]) compared to results from the present study (3017 mg kg^−1^). Additionally, Abukawsar et al. found a higher value of Ca in two cultivars of black pepper, 6280 mg kg^−1^ and 9230 mg kg^−1^, [12]. For cayenne pepper, Hwang et al. determined a lower value of Ca in a range from 701 mg kg^−1^ to 1372 mg kg^−1^ [21], and Zhang et al. determined a value ranging from 963 mg kg^−1^ to 1480 mg kg^−1^ [9], compared with the obtained result of 2290 mg kg^−1^. Ethiopian red pepper showed a completely different pattern for macro- and microelements, with all data being lower than those determined in the present study except for Fe and Zn [22], which are common contaminants in inorganic trace laboratories. This fact might be due to the different growing areas and/or different mixture of chili pepper species.

The Na content in white, green and black pepper was also considerable (10.8 mg kg^−1^–73.7 mg kg^−1^), presenting a similar value to that of Savić and coworkers (67.39 mg kg^−1^, [20]) and a lower value compared with results of Abukawsar et al. (155 mg kg^−1^ and 162 mg kg^−1^, [12]). In the present study, cayenne pepper was found to have a higher content of Na (385 mg kg^−1^) compared to the results of Savić et al. (57.7 mg kg^−1^, [20]) but significantly lower than those obtained by Hwang et al. (1100 mg kg^−1^–3812 mg kg^−1^, [21]).

Magnesium was found in a range from 750 mg kg^−1^ to 2382 mg kg^−1^, similar to Abukawsar et al.’s results (2330 mg kg^−1^ and 2340 mg kg^−1^, [12]), but lower than the results of Savić et al. (4.08 mg kg^−1^–34.12 mg/kg^−1^, [20]) and Hwang et al. (1554 mg kg^−1^–1919 mg kg^−1^, [21]). 

The contents of microelements, on the other hand, varied more noticeably in the pepper samples as a function of the species. There was a high content of Cr, Ga, Ni and Ag, with the highest for Ag in green (3.99 mg kg^−1^) and white pepper (2.98 mg kg^−1^), and lowest for black pepper (0.133 mg kg^−1^). Abukawsar et al. measured a lower amount of Ag in two black pepper cultivars, namely 0.00654 mg kg^−1^ and 0.018 mg kg^−1^, [12].

The presence of Cr was detected in black, white and green pepper 1.587 mg kg^−1^, 2.001 mg kg^−1^ and 1.822 mg kg^−1^, respectively, though it was lower than that published by Abukawsar et al. for black pepper 0.266 mg kg^−1^ and 0.401 mg kg^−1^, [12]. In cayenne pepper, the content of Cr is 2.693 mg kg^−1^ is similar to the maximum value presented by Zhang et al. (0.338 mg kg^−1^–2.21 mg kg^−1^, [9]) and lower than that reported by Hwang et al. (0.856 mg kg^−1^–0.993 mg kg^−1^, [21]). 

The content of Ga found in this study was between 0.30 mg kg^−1^ and 2.22 mg kg^−1^, which was more than that reported by Abukawsar et al. for black pepper (0.021 mg kg^−1^ and 0.046 mg kg^−1^, [12]). In cayenne pepper, Hwang et al. found smaller amounts of Ga (0.030 mg kg^−1^–0.060 mg kg^−1^, [21]) compared to the obtained results of 0.30 mg kg^−1^. 

The presence of Ni in pepper samples was found to range from 0.320 mg kg^−1^ to 1.699 mg kg^−1^. In cayenne pepper, the content of Ni is 0.720 mg kg^−1^, which is similar to the values reported by Hwang et al. (0.637 mg kg^−1^–1.03 mg kg^−1^, [21]) and Zhang et al. (0.729 mg kg^−1^–2.73 mg kg^−1^, [9]). Increased Ni supply, e.g., from soil or irrigation water, was found to cause elevated Ni contents in the leaves and fruits of *Capsicum annuum*, but to reduce the contents of the essential elements N, P, and K [23].

In all pepper samples, the mean content of Mo is 0.218 mg kg^−1^, and in cayenne pepper, it is 0.485 mg kg^−1^, which is equal to the mean value obtained by Zhang et al. (0.130 mg kg^−1^–1.33 mg kg^−1^). 

The presence of Li was found only in cayenne pepper (1.356 mg kg^−1^) and is higher compared to the results of Savić et al. (0.26 mg kg^−1^, [20]) and Hwang et al. (0.072 mg kg^−1^–0.629 mg kg^−1^, [21]). For other pepper samples, the values for Li were below the LOD (0.016 mg kg^−1^), or at least lower than the results reported by Savić et al. for black pepper (0.13 mg kg^−1^, [20]) and Abukawsar et al. (0.179 mg kg^−1^ and 0.221 mg kg^−1^, [12]). 

Results for the Co content in black pepper (0.027 mg kg^−1^) are similar to those reported in literature data, including those reported by Abukawsar et al. (0.043 mg kg^−1^ and 0.052 mg kg^−1^, [12]). The result for Co content in this study in cayenne pepper (0.244 mg kg^−1^) is similar to the data reported by of Zhang et al. (0.137 mg kg^−1^–0.346 mg kg^−1^, [9]) and Hwang et al. (0.126 mg kg^−1^–0.175 mg kg^−1^, [21]). 

Lower values were found for Se in cayenne pepper (0.006 mg kg^−1^) compared to those reported by Hwang et al. (0.054 mg kg^−1^–0.134 mg kg^−1^, [21]) and in black pepper, 0.034 mg kg^−1^, compared to Abukawsar et al.’s results (0.684 mg kg^−1^ and 1.175 mg kg^−1^, [12]). 

Savić et al. [20] found a slightly higher amount of Bi in black pepper 0.59 mg kg^−1^ compared to the obtained value of 0.22 mg kg^−1^ and to Abukawsar et al.’s values (0.00748 mg kg^−1^ and 0.0101 mg kg^−1^, [12]). In cayenne pepper, Bi was found in a smaller amount (0.04 mg kg^−1^) compared to Savić et al.’s results (2.19 mg kg^−1^, [20]). 

In the current study, the content of Be was found to be below the LOD (0.00094 mg kg^−1^). Abukawsar et al. [12] found Be (0.0063 mg kg^−1^) in black pepper, green pepper (0.003 mg kg^−1^) and cayenne pepper (0.006 mg kg^−1^).

The content of V in black pepper (0.024 mg kg^−1^) is lower compared to Abukawsar et al.’s results (0.078 mg kg^−1^ and 0.299 mg kg^−1^, [12]), but higher in cayenne pepper (0.361 mg kg^−1^), [12] compared to Hwang et al.’s results (0.012 mg kg^−1^–0.094 mg kg^−1^).

Except the results for Tl of Zhang et al. in cayenne pepper (0.032 mg kg^−1^–0.044 mg kg^−1^, [9]), all other results for Tl were below LOD. For Te content in black pepper, no literature data are available for comparison with the data obtained in the present investigation (0.0024 mg kg^−1^–0.361 mg kg^−1^).

The content of Pb in black pepper (0.071 mg kg^−1^) was similar to Zheng et al.’s findings (0.056 mg kg^−1^–0.210 mg kg^−1^, [9]) but lower compared to the results of Savić et al. (0.13 mg kg^−1^, [20]) and Abukawsar et al. (0.159 mg kg^−1^, [12]). Hwang et al. found Pb in cayenne pepper in a smaller range, 0.031 mg kg^−1^–0.087 mg kg^−1^, [21], compared to the obtained value (0.134 mg kg^−1^).

In black pepper, As was below LOD (0.00061 mg kg^−1^) but Abukawsar et al. found As ranging from 0.001328 mg kg^−1^ to 0.004854 mg kg^−1^, [12]. In cayenne pepper, As was found in a higher amount (0.145 mg kg^−1^) compared to values of Hwang et al. (0.034 mg kg^−1^–0.076 mg kg^−1^, [21]) and Zhang et al. (0.039 mg kg^−1^–0.085 mg kg^−1^, [9]). 

For black pepper, the content of Cd (0.009 mg kg^−1^) is similar to the values obtained by Abukawsar et al. for two different samples (0.011 mg kg^−1^ and 0.00477 mg kg^−1^) [12]. A higher amount of Cd was found in cayenne pepper (0.069 mg kg^−1^), which is similar to the values reported by Hwang et al. (0.072 mg kg^−1^–0.093 mg kg^−1^, [21]) but lower compared to Zhang et al.’s results (0.175 mg kg^−1^–0.382 mg kg^−1^, [9]).

In black pepper, Cu was found to a higher extent, namely 10.7 mg kg^−1^, compared to the values measured by Savić et al. (2.58 mg kg^−1^, [20]). Compared to the obtained result for Cu (7.252 mg kg^−1^) in cayenne pepper, Hwang et al. found less (5.692 mg kg^−1^–6.983 mg kg^−1^, [21]) and Zhang et al. more (9.93 mg kg^−1^–15.0 mg kg^−1^, [9]) Cu.

In the studied pepper samples, Zn was present in a higher amount for black pepper (15.0 mg kg^−1^) compared to the results of Savić et al. (3.26 mg kg^−1^, [20]. In cayenne pepper (17.45 mg kg^−1^), the amount of Zn is similar to Hwang et al.’s results (16.4 mg kg^−1^–19.8 mg kg^−1^, [21]) but smaller than the values reported by Zhang et al. (17.9 mg kg^−1^–37.9 mg kg^−1^, [9]). 

In cayenne pepper, a similar content of Rb (15.86 mg kg^−1^) was found compared to those reported by Zhang et al. (17.3 mg kg^−1^–22.3 mg kg^−1^, [9]) and Hwang et al. (6.61 mg kg^−1^–17.5 mg kg^−1^, [21]). 

A lower amount of Se in black pepper (0.61 mg kg^−1^, [20]) was found by Savić et al. compared to 18.3 mg kg^−1^. In cayenne pepper, the results for Se (0.034 mg kg^−1^–0.124 mg kg^−1^) were lower compared to the values obtained by Hwang et al. (1.30 mg kg^−1^–4.26 mg kg^−1^, [21]) and Zhang et al. (3.11 mg kg^−1^–11.4 mg kg^−1^, [9]). 

In black pepper, compared to the result for Ba (30.2 mg kg^−1^), all literature data are lower than those reported Savić et al. (1.12 mg kg^−1^, [20]) and Hwang et al. (0.613 mg kg^−1^–2.02 mg kg^−1^, [21]). 

In this study, the obtained value for Mn (46.8 mg kg^−1^) in pepper was much higher compared to the value reported by Savić et al. (1.12 mg kg^−1^, [20]). Similar values for Mn (16.6 mg kg^−1^) in cayenne pepper were found by Hwang et al. (10.7 mg kg^−1^–14.7 mg kg^−1^, [21]) and Zhang et al. (14.9 mg kg^−1^–21.8 mg kg^−1^, [9]). 

In black pepper, as well as in white and green, similar values were found for Fe (23.0 mg kg^−1^–26.4 mg kg^−1^), though these were higher than the values for Fe in black pepper measured by Savić et al. (2.69 mg kg^−1^, [20]) and smaller compared to Abukawsar et al.’s results (138 mg kg^−1^ and 344 mg kg^−1^, [12]). In cayenne pepper, a lower amount of Fe was found (230 mg kg^−1^) compared to Hwang et al.’s (1.16 mg kg^−1^–1.21 mg kg^−1^, [21]) and Zhang et al.’s results (57.4 mg kg^−1^–166 mg kg^−1^, [9]). 

Regarding the toxicological assessment of plant-derived foods, only the mass fractions of As, Cd and Pb in raw (medical) plant material is limited by the World Health Organization (WHO), with the maximum permissible values being 1.0 mg/kg, 0.3 mg/kg and 10 mg/kg, respectively [24]. Based on these prescribed contents, it can be seen that the analyzed pepper samples are not supposed to be for use and consumption.

For the present study, commercially available pepper samples where used, which lacked the information on specific cultivars as well as exact growing area, as only the country India was given. Whilst the secondary metabolic profile was found to be cultivar-dependent [25], the differences for metals based on only two cultivars were not significant [12]. An investigation of hot chili originating from Thailand revealed lower contents for the major elements (esp. K) [26] than those obtained for the cayenne pepper samples analyzed in the present study.A group of Italian researchers analyzed unprocessed chili pepper samples from different regions and found that when it comes to samples from India, the elemental composition is widespread and not as unique as for smaller areas such as Calabria [27]. 

### 3.3. Chemometric Analysis

Since commercially available products were used, which lacked information regarding the growing area, soil composition, climatic conditions and a detailed description of their processing, these influencing parameters have not been included in the statistical evaluation in this study.

**Figure 2:**
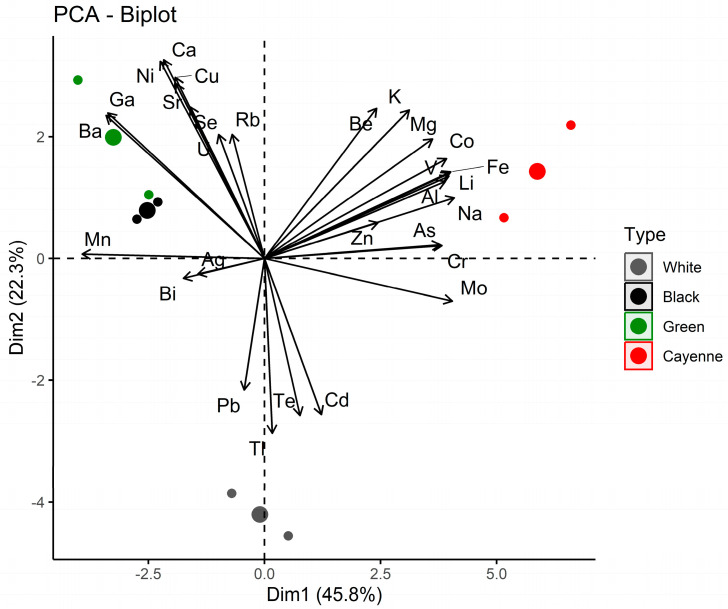
PCA biplot.

The dataset consisting of 8 samples and 29 elements (variables) underwent a PCA and an HCA. The PCA of the first two dimensions in Figure 2 explains 68% of the variation. The biplot shows that the variables, as the drivers to the location of the samples, are located in all four quartiles, with the majority of them in the first and second quartiles. The contributions of the elements to PC1 (Na, Mo, Fe, Li, V, Mn, Mo, Cr, As, Ba, Ga, K) and PC2 (Ca, Ni, Cu, Tl, Sr. Te, Cd, Se, Be, K, Ga, Ba, Pb), as well as their combined contribution, are presented in Figure S1 in the supplementary information. Figure S2 shows the location of the eight samples (individuals) in the PCA. The samples were well-distinguished, with the two cayenne peppers located in the first quartile, and green and black peppers in the second quartile. The two white pepper samples were located in the third and fourth quartiles, very close to the origin of PC1.

**Figure 3:**
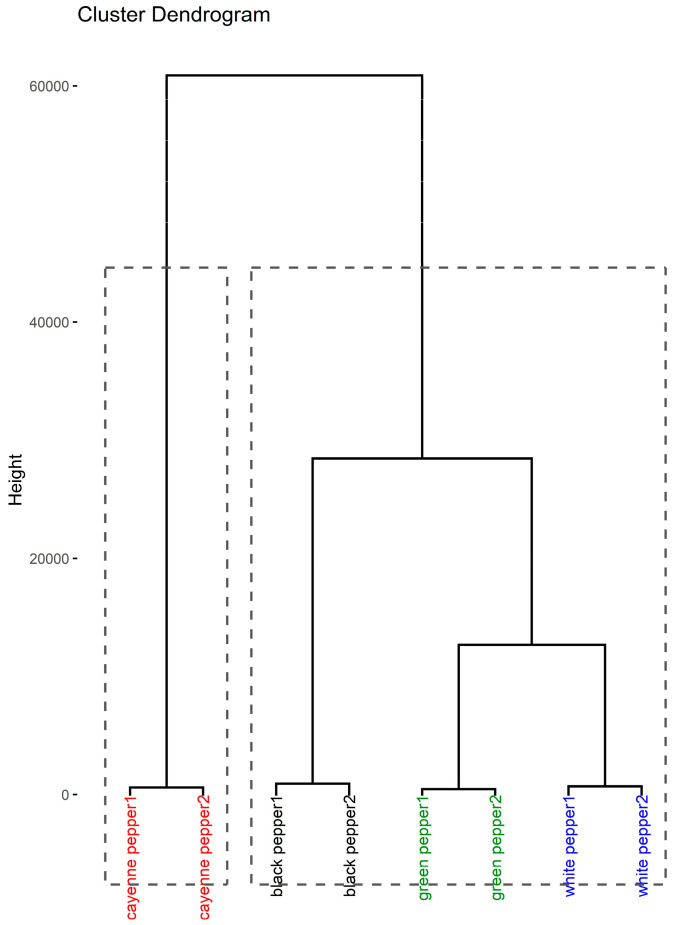
Dendrogram of hierarchical cluster analysis.

The HCA is depicted as a dendrogram in Figure 3. Two distinct clusters were formed, whereby the first cluster consisted of the two cayenne pepper samples and the second contained pairwise subclusters of black peppers, green peppers, and white peppers. 

Despite the very distinct biplots and clusters, the statistical analysis did not identify significant differences between the samples or the sample types. The Kruskal–Wallis *p*-value was 0.71 and the Benjamini–Hochberg was *p* = 0.80 between all types.

**Figure 4:**
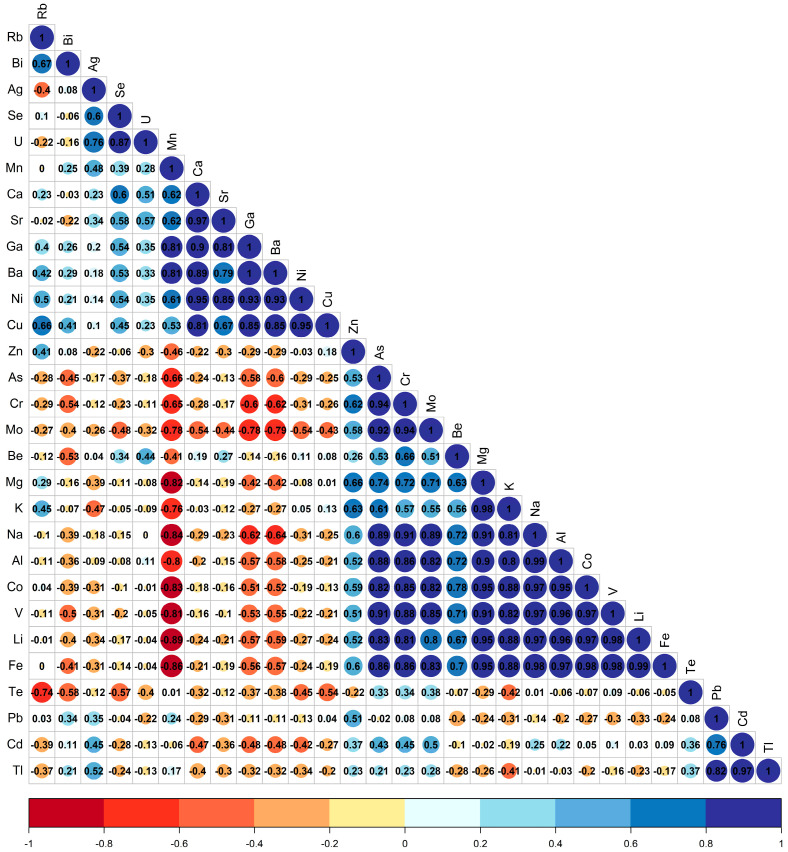
Correlation between the elements.

The Pearson correlation between elements using Euclidean distances and the Ward.D2 method (Figure 4) shows negative (red color) and positive (blue color) correlations. Elements from Zn to Te on the y-axis are negatively correlated with elements from As to Te on the x-axis. Within both clusters, some pairs are highly correlated with r > 0.75. Additionally, the elements Ga, Ba, Ni, Cu and Zn are highly correlated among each other. A high correlation between Al and Fe in spices has been reported in the literature [28,29]. Correlations for selected pairs of analytes are plotted in the supplementary information (Figure S3) to show the significance of the correlations found. 

In order to evaluate and find correlations between elements in the given context, they need to be classified for biological significance. In a technical report by IUPAC, Duffus [30,31] has categorized the elements of the periodic table according to the last electron shell into s-, p-, d-, f-block elements (later referred to as “Block”), based on Lewis acid behavior into A (hard), B (soft), and borderline (BL) (later referred to as “Class”), and in geochemical classification as lithophile or chalcophile (later referred to as Geochem). It should be mentioned that not all the elements analyzed have been categorized by Duffus or later by Appenroth [32] as regards their importance in plant sciences. In plant science, metals or metalloids do not matter in their elemental form; however, their ionic forms are relevant, which is related to the position of the elements in the periodic system and their chemical physical properties.

**Figure 5:**
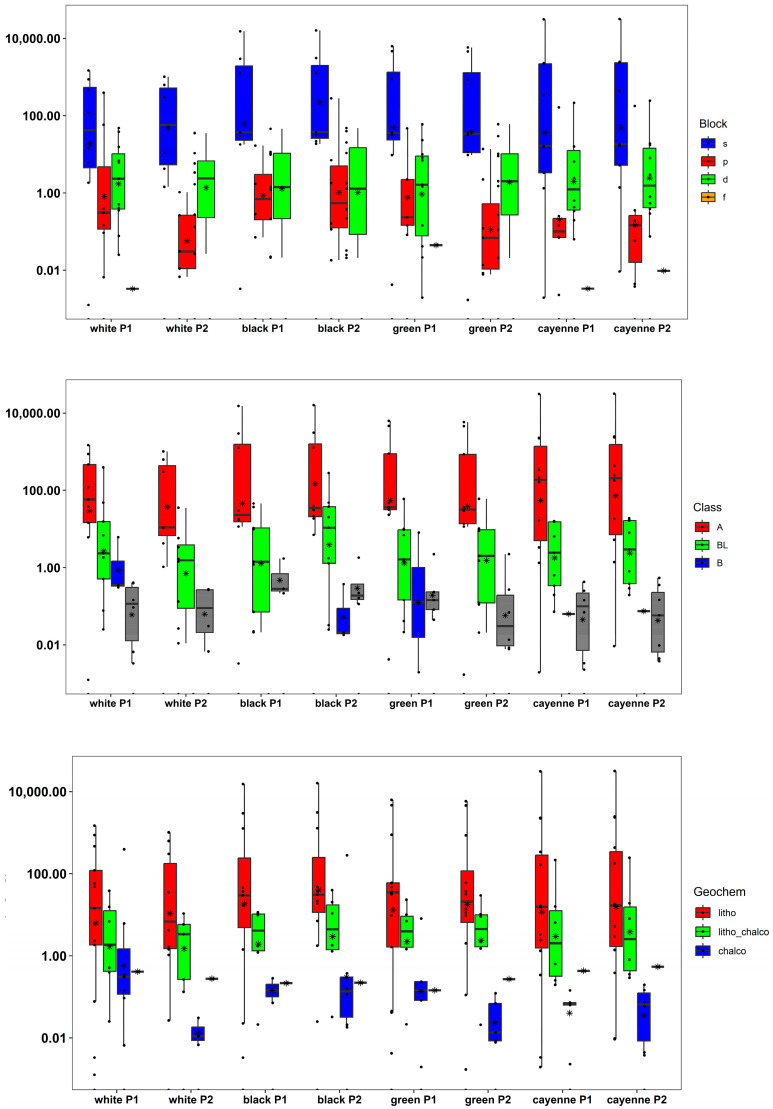
Abundance of analytes grouped according to Block, Class or Geochem (box whisker); y-axis in all cases mass of fraction in mg kg^−1^, where grey color in the boxes indicates that the element was not assigned to any of the “Class” or “Geochem”; see also “NA” in Figure 6. The whiskers represent the minimum and maximum concentrations without the outliers. The lower border of the box represents the first quartile (25%), the line inside the box the median and the upper border is the third quartile (75%). The mean is represented as (X). The dots outside the whiskers are outliers, which were defined as all concentrations greater or smaller the interquartile range multiplied by 1.5.

**Figure 6:**
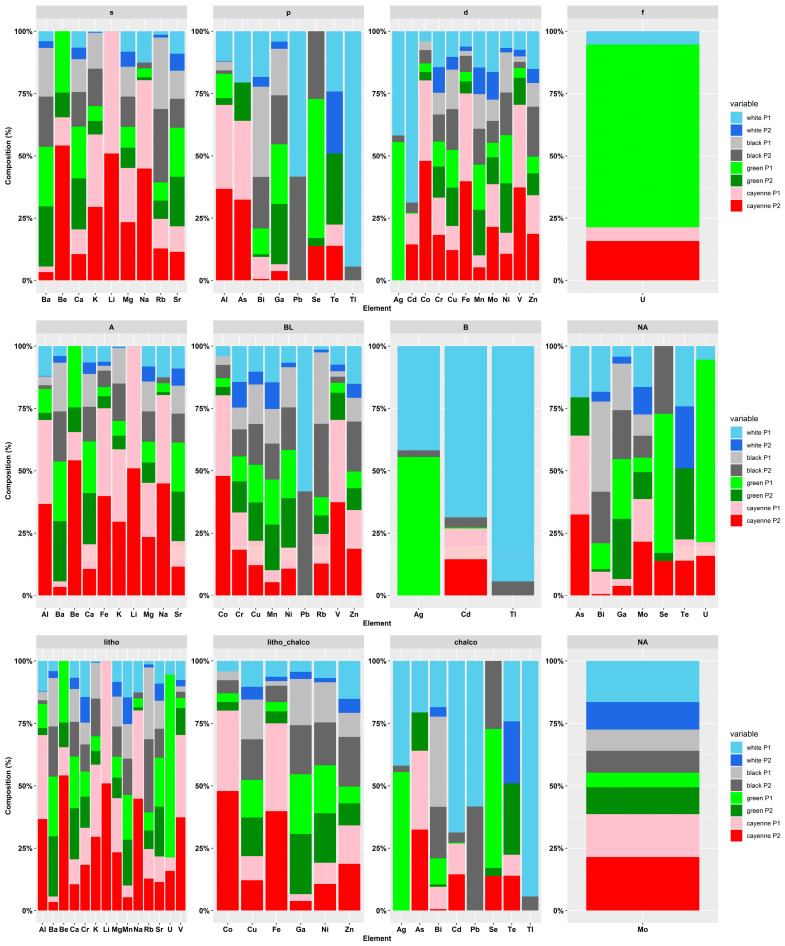
Elemental pattern according to Block, Class or Geochem NA indicates that the element was not assigned to any of the “Class” or “Geochem”; see also “grey color” in Figure 5.

**Figure 7:**
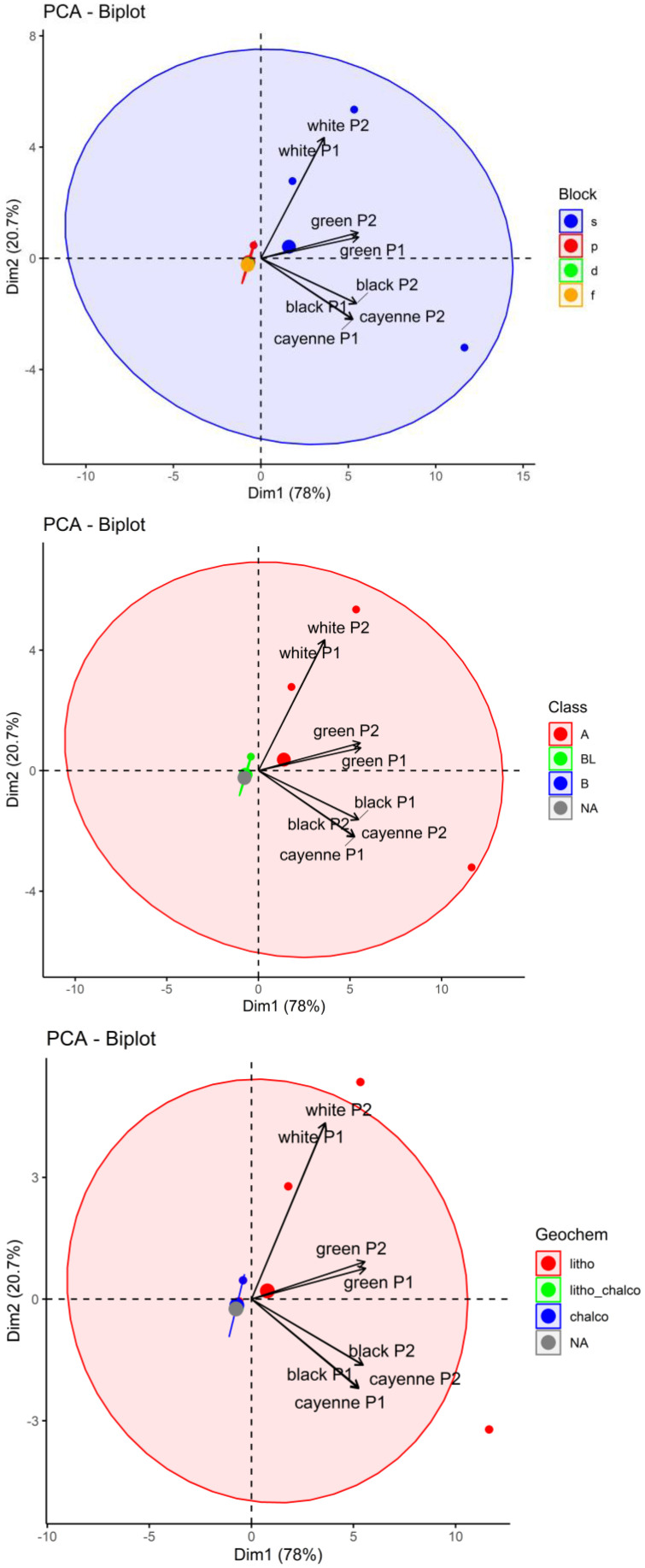
PCA with ellipses according to Block, Class or Geochem. NA indicates that the element was not assigned to a “Class” or “Geochem”.

The following figures (Figure 5, Figure 6 and Figure 7) show certain discriminations of the elements in the analyzed six pepper samples using the IUPAC approach. It can be seen that the s-block elements dominate in all pepper samples. These results coincide well with the Lewis acid properties of hard acids (class A). With respect to the geochemical classification, a differentiation was observed, and it was found that the white pepper samples had higher percentages of chalcophile elements than the other three types of peppers, especially for Cd Pb, Te, and Tl. The lithophile elements showed a greater abundance in the cayenne pepper, especially for Al, Be, K, Li, Na, and V.

## 4. Conclusions

The results obtained in the present investigation enhance the data on the element composition of different pepper types originating from India and commercially available in Croatia, especially in relation to the number of elements analyzed, as there is a scarcity of papers on this topic. The information regarding macro-, micro-, and trace elements attained proved their beneficial contribution to human diet, and revealed the values associated with a safe use concerning the content of harmful elements.

The significantly different pattern found for macro- as well as microelements can be a promising tool to distinguish different types of pepper from one region (i.e., India in the present study), which can help to prove fraud and authentication. Apart from the species-specific uptake and accumulation behavior, the further processing of the pepper fruits strongly influences the final elemental composition of the respective spice. The detailed statistical evaluation showed that not all analyzed elements contribute to the classification of pepper types or are highly correlated, which allows for a reduction in analytes and data volume in following investigations.

To further improve the presented method, more influencing parameters, such as growing area, soil composition, climatic conditions and a detailed description of the pepper fruits’ processing, will be considered in the study design of future experiments.

## Data Availability

Data is contained within the article or Supplementary Material.

## Supplementary Materials

The following supporting information can be downloaded at: https://www.mdpi.com/article/10.3390/foods12163132/s1, Tables S1 and S2: Mean mass fraction of macro- and microelements (n = 6) and LOD in mg/kg, alongside precision and recovery in %. Figure S1: Contributions of variables (elements) to the PCA (above). Dim1 (middle). and Dim2 (below) The red dotted line indicates the average contribution. Figure S2: Location of the samples and contribution to the individuals. Figure S3: Correlation for selected pairs of analytes.
