# Supplementary material for: Preliminary Study of Pepper Types Based on Multielement Content Combined with Chemometrics
Source: Foods. 2023 Aug 21;12(16):3132. doi: 10.3390/foods12163132 (PMC10453101; doi:10.3390/foods12163132)
Supplement: Supplementary file 1 [file foods-12-03132-s001.zip › foods-2488711-supplementary.pdf]

Table S1. Mean mass fraction of macroelements (n = 6) and LOD in mg/kg alongside precision and recovery in %.

Analyte	White pepper	Black pepper	Green pepper	Cayenne pepper	LOD [14]	Precision [14]	Recovery [14]
Ca	1252	3017	4614	2290	0.15	1.5	113
Mg	750	1273	875	2382	0.12	1.2	102
Na	60,3	10,8	23,3	385	1.9	0.5	103
K	383	15742	6092	31359	0.6	1.3	93

Table S2. Mean mass fraction of microelements (n = 6) and LOD in mg/kg, alongside precision and recovery in %.

Analyte	White pepper	Black pepper	Green pepper	Cayenne pepper	LOD [14]	Precision [14]	Recovery [14]
**Ag**	2.98	0.133	<LOD	3.99	0.0042	3.9	107
**Al**	29.5	26.9	170	170	0.017	1.3	82
**As**	0.043	<LOD	0.145	0.011	0.00061	3.6	98
**Ba**	5.14	30.2	4.27	36.7	0.0034	1.3	86
**Be**	<LOD	<LOD	0.006	0.006	0.0057	2.3	87
**Bi**	0.09	0.22	0.04	0.05	0.00094	3.7	91
**Cd**	0.173	0.009	0.069	0.003	0.0007	1.5	86
**Co**	0.013	0.027	0.021	0.0244	0.0042	1.4	84
**Cr**	2.00	1.59	2.69	1.82	0.0015	3.6	115
**Cu**	5.10	10.7	7.25	10.1	0.0084	0.7	89
**Fe**	24.5	25.6	23.0	26.4	0.061	0.6	109
**Ga**	0.328	1.77	0.30	2.22	0.0068	0.6	102
**Li**	<LOD	<LOD	1.356	<LOD	0.016	2.2	82
**Mn**	41.6	46.8	16.6	59.9	0.0032	1.1	84
**Mo**	0.343	0.218	0.485	0.207	0.0072	3.8	113
**Ni**	0.320	1.25	0.720	1.47	0.0024	1.4	111
**Pb**	0.011	0.071	0.134	0.133	0.0016	1.5	89
**Rb**	1.6	37.3	15.86	9.5	0.0022	1.3	95
**Se**	<LOD	0.034	0.006	0.124	0.049	4.0	115
**Sr**	12.8	18.3	17.5	31.9	0.0036	1.1	90
**Te**	0.007	<LOD	0.003	0.004	0.00012	2.6	87
**Tl**	<LOD	<LOD	<LOD	<LOD	0.0035	2.0	92
**V**	0.052	0.024	0.361	0.076	0.0041	2.2	104
**Zn**	10.6	15.1	17.45	7.98	0.011	1.4	114

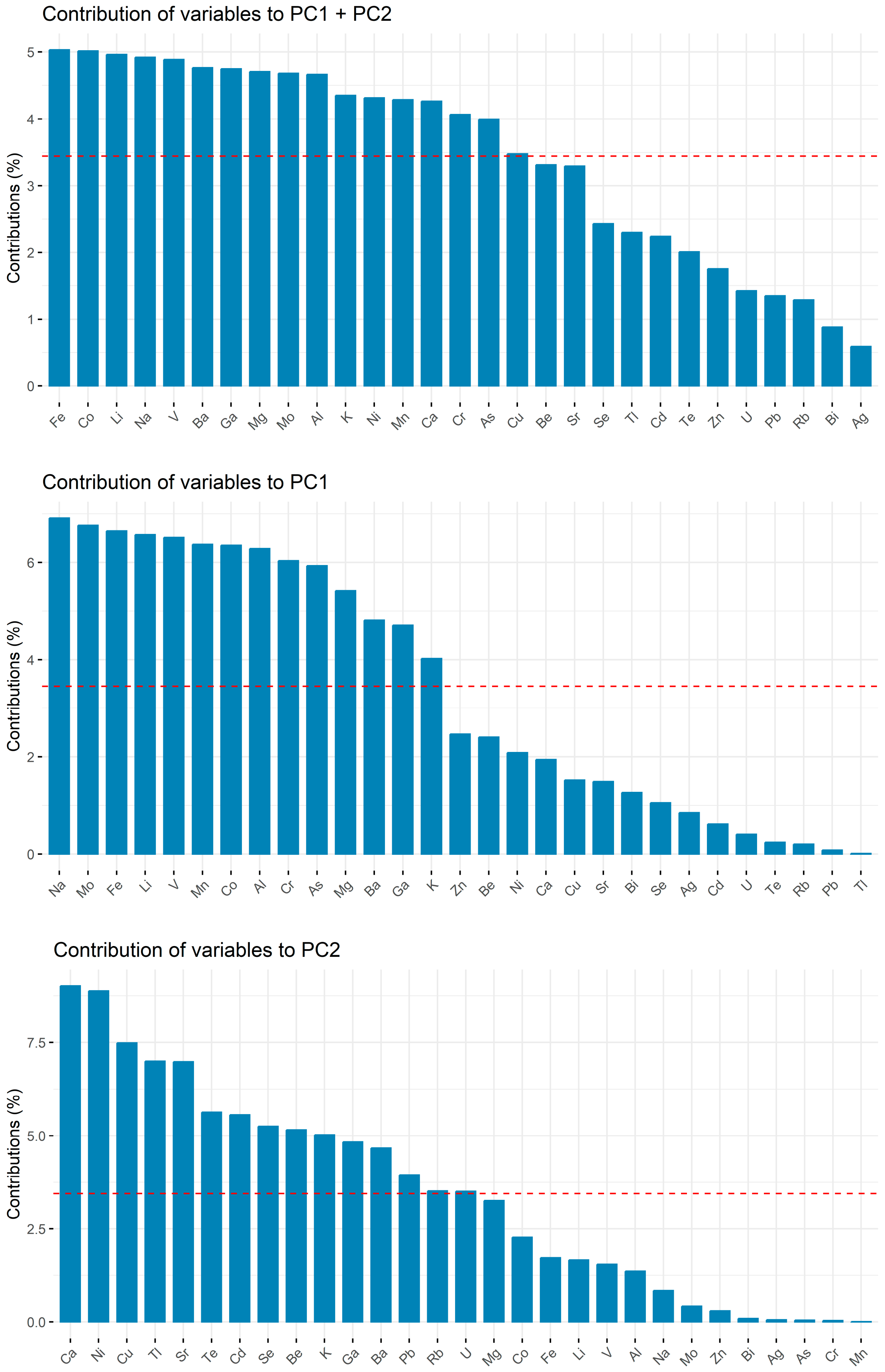

Figure S1. Contributions of variables (elements) to the PCA (above). Dim1 (middle). and Dim2 (below) The red dotted line indicates the average contribution.

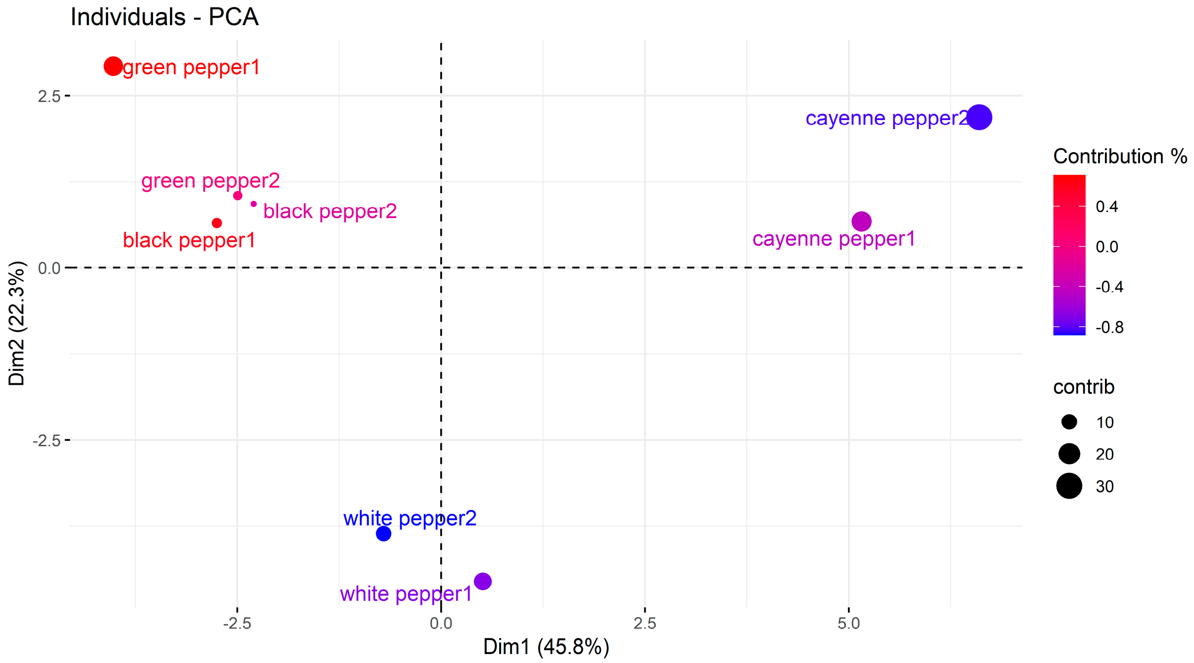

Figure S2. Location of the samples "(individuals) in the PCA" and contribution to the individuals.

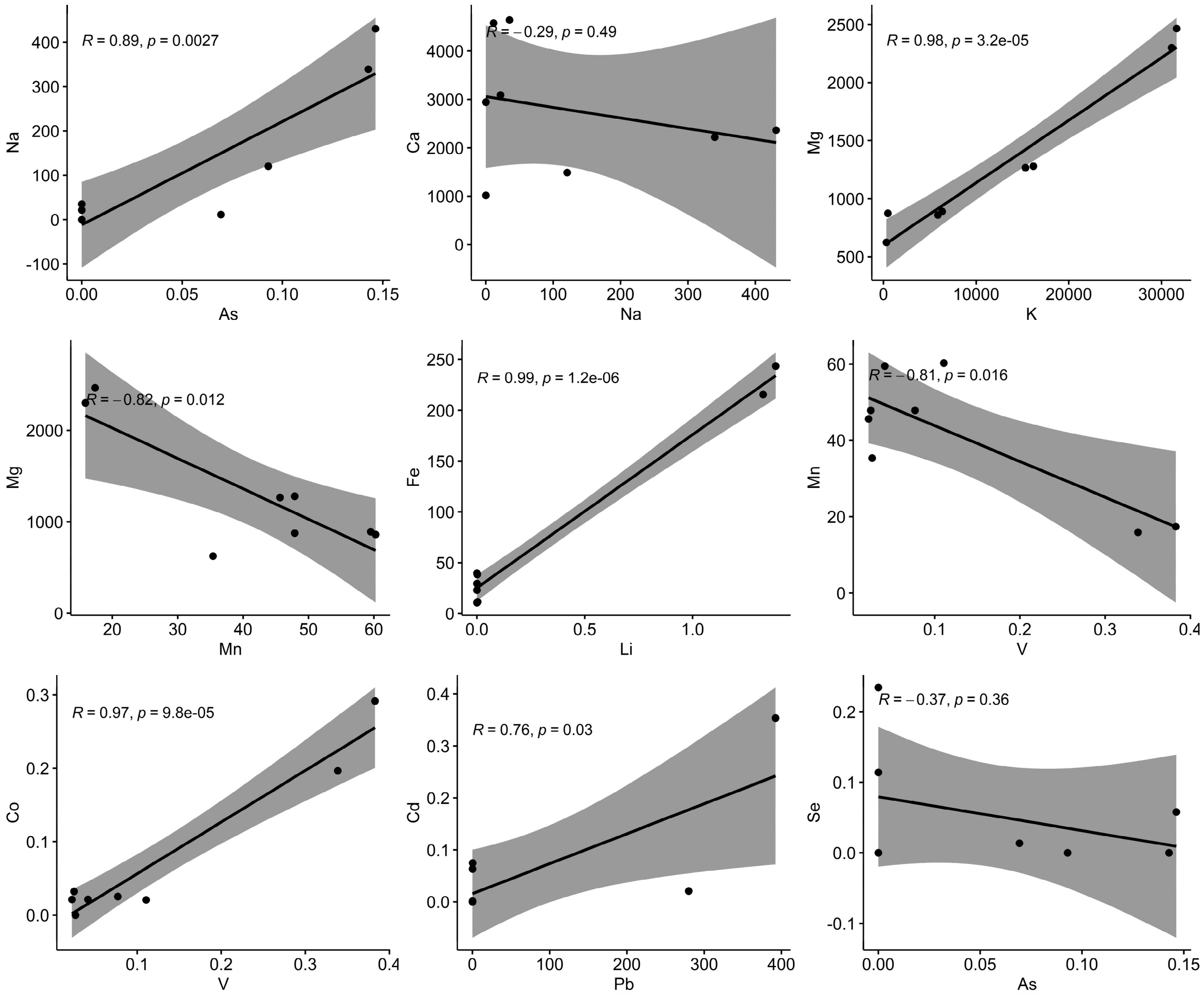

Figure S3. Correlation of selected pairs of analytes.
